# Investigation and control of an outbreak of urinary tract infections caused by *Burkholderia cepacia*n-contaminated anesthetic gel

**DOI:** 10.1186/s13756-020-00855-x

**Published:** 2021-01-06

**Authors:** Mingmei Du, Linjian Song, Yan Wang, Jijiang Suo, Yanling Bai, Yubin Xing, Lijun Xie, Bowei Liu, Lu Li, Yanping Luo, Yunxi Liu

**Affiliations:** 1 Department of Disease Prevention and Control, First Medical Center of Chinese PLA General Hospital, Beijing, China; 2 Center for Clinical Laboratory Medicine, First Medical Center of Chinese PLA General Hospital, Beijing, China; 3 Department of Urology, First Medical Center of Chinese PLA General Hospital, Beijing, China

**Keywords:** *B. cepacia*, Outbreak, Urinary tract infection

## Abstract

**Background:**

This report describes an outbreak of 71 patients developed *B. cepacia* urinary tract infection (UTI) by contaminated single-use anesthetic gel.

**Methods:**

Epidemiological investigation of patients with *B. cepacia*-positive urine or blood samples between March 19, 2018 and Novemeber 15, 2018 was conducted to identify the source of infection. Microbiological samples from hospital surfaces, endoscopes, disposable items, and the hands of staff were tested for *B. cepacia* contamination. Pulsed-field gel electrophoresis (PFGE) was used to compare homology in *B. cepacia* isolates.

**Results:**

During the outbreak, nosocomial *B. cepacia* UTI was confirmed in 71 patients. Epidemiological investigation showed that 66 patients underwent invasive urological diagnosis and treatment, while the remaining five patients underwent bedside indwelling catheterization, with all patients exposed to single-use anesthetic gel. All batches of anesthetic gel were recalled and the outbreak abated. Overall, 155 samples were collected from environmental surfaces and disposable items, and *B. cepacia* contamination was confirmed in samples from one used cystoscope and three anesthetic gels from the same batch. PFGE showed homology between 17 out of 20 *B. cepacia* isolates from patients and three isolates from the contaminated anesthetic gel. All patients achieved cure.

**Conclusion:**

Contaminated single-use anesthetic gel was confirmed as the source of the *B. cepacia* outbreak, with infection occurring during invasive urological diagnostic and treatments. Thus, investigations of nosocomial outbreaks of *B. cepacia* infection should consider contamination of diagnostic and treatment items used in infected patients.

## Background

*Burkholderia cepacia* is a Gram-negative, aerobic, non-fermentative bacterium that is widely distributed in the hospital environment. It is also a opportunistic pathogen in immune-impaired patients with cystic fibrosis or chronic granulomatosis [1]. In recent years, there have been frequent nosocomial outbreaks of respiratory tract, blood, and urinary tract infection (UTI) caused by *B. cepacia*-infected ward water sources, oral liquid, ultrasound gel, skin disinfectant, and mouthwash [2–6]. In 2012, Sutton and Jimenez published a summary of product recalls by the Food and Drug Administration involving microbial contamination [7]. They showed that between 2004 and 2011, *B. cepacia* contamination was associated with 34% of non-sterile product recalls. In the current study, we report an outbreak of UTI among 71 patients in a tertiary general teaching hospital in China that occurred between March 2018 and November 2018. Epidemiological and microbiological investigations confirmed that the outbreak was caused by a batch of *B. cepacia*-contaminated single-use anesthetic gel, which was used for anesthesia and lubrication of the urethra during invasive diagnosis and treatment. The outbreak abated after the hospital stopped using the product.

## Methods

### Hospital setting

The tertiary general teaching hospital where the outbreak occurred has a total of 3800 beds, with ~ 120 beds across the four inpatient wards of the Urology Surgery Department. Most urological invasive diagnoses and treatments (mainly for the examination of urinary diseases, biopsies, and minor operations) are accomplished in the outpatient cystoscope room, with about 500 cases seen per month. The outpatient cystoscope room has an adjacent room that is used for cleaning and disinfection of flexible and rigid cystoscopes.

### Case definition

Diagnosis of UTI was performed according to the Chinese Nosocomial Infection Diagnostic Criteria (2001) [8], as follows:

*UTI*: patients have symptoms of urinary tract irritation such as frequent urination, urination urgency, and pain on urination, or have lower abdominal tenderness, kidney area percussion pain, with or without fever, urine culture or blood culture is *B. cepacia*-positive. Patients also have one of the following:

1. Urine has ≥5 white blood cells/high power field of vision in males and ≥ 10 white blood cells/high power field of vision in females.
2. Diagnosed as UTI by clinician, or UTI identified by effective antibacterial treatment.

For inpatients with *B. cepacia* in urine or blood samples, non-nosocomial infection was defined as *B. cepacia* identification within 48 h of admission, while nosocomial infection was defined as *B. cepacia* identification later than 48 h post-admission. If infection was directly related to the last outpatient or inpatient diagnosis and treatment, the case was defined as nosocomial infection regardless of the identification time.

### Epidemiological investigation

On August 21, 2018, doctors in the Third Ward of the Urology Department reported an upward trend in the detection of *B. cepacia* in the urine of patients. The *B. cepacia* detection data for the ward from January to August 2018 were collected, and showed 11 cases of *B. cepacia* infection between April 30, 2018 and August 08, 2018. In eight cases, *B. cepacia* was detected within 48 h of admission, indicating that the patients were infected prior to admission. The three cases of nosocomial *B. cepacia* infection occurred in three different months, indicating that they did not constitute an outbreak of nosocomial infection. Instead, the infections were thought to be caused by *B. cepacia* cross-contamination from non-nosocomial infection patients via cystoscopy or surgical treatment equipment.

On September 13, 2018, the investigation was expanded to include inpatients across the entire hospital, focusing on epidemiological investigations of patients with *B. cepacia*-positive urine and/or blood samples. Detailed invasive examination and operation records for both outpatients and inpatients of the Urology Department were examined. The analysis showed that most *B. cepacia*-positive patients were identified within 48 h of first admission, and had previously undergone invasive diagnosis and treatment in the outpatient cystoscope room of the studied hospital. As a result, 25 patients initially classified as non-nosocomial infection were revised to nosocomial infection. Data for the infected patients was re-investigated and showed that by September 30, 2018, 58 patients who met the case definition were distributed across the urology (38 cases), gynecology (10 cases), and other (10 cases) departments, and that all patients underwent invasive operations or urological examination. Importantly, only four *B. cepacia*-positive cases (detected in urine or blood) were recorded over the same time period in 2017. Thus, the cluster of cases was confirmed to be an outbreak.

### Microbiological sampling and identification

Microbiological samples were collected from possible exposure surfaces, endoscopes, disposable items, and the hands of staff and screened for *B. cepacia* contamination used sterile swabs with sterile saline solution. Microbiological sampling was carried out a total of four times during the outbreak investigation. Samples included: 20 samples from cystoscopes, environmental surfaces, and the hands of doctors and nurses from the outpatient cystoscope room, collected on August 23, 2018; 70 samples from rigid and flexible cystoscopes, environmental surfaces, and the hands and clothing of staff from the Third Ward of the Urology Department. From November 11–15, 2018, the cystoscope sampling method was replaced with routine irrigation and disposable brushing, and 27 samples were collected from cystoscopes cleaned using different disinfection methods, while 20 samples were collected from disposable items (including one anesthetic lubrication gel, batch 20,170,521). A total of 18 samples were collected from all five batches of anesthesia gel and four batches of coupling agent on November 23, 2018.

All bacteria were isolated from microbiological samples according to the National Clinical Inspection Operation specifications. Species identification and in vitro antibiotic susceptibility tests were carried out using the Vitek II system (bioMérieux, Marcy-l’Étoile, France) following the 2018 breakpoints defined by the Clinical and Laboratory Standards Institute (CLSI) [9]. Molecular typing by pulsed-field gel electrophoresis (PFGE) was performed for 24 *B. cepacia* isolates, including 20 isolates from nosocomial infection patients, one isolate from a non-nosocomial infection patient, and three isolates from the anesthetic gel. In addition, the B cepacia strain SperI (40 U) was used, and the Salmonella standard strain H9812 was digested with XbaI (40 U) at 37 °C for > 3 hs. The following electrophoresis conditions were used: voltage, 6 V/cm; pulse time, 5.0–50.0 s; linear conversion; conversion angle, 120°; electrophoresis time, 20 hs; and electrophoresis temperature, 14 °C. CHEF III (Bio-Rad, USA) was used for PFGE.

### Intervention measures

Different intervention measures were taken at different stages of the outbreak investigation, as follows:

1. Intervention A: from August 21, 2018, cleaning and disinfection of the surfaces of objects in the outpatient cystoscope room was strengthened, the hand hygiene and aseptic operation of staff was standardized, and the cleaning and disinfection of the endoscopes in the outpatient cystoscope room was supervised.
2. Intervention B: from September 13, 2018, the endoscopes in the outpatient cystoscope room were sent to the disinfection center each day after the last time of use, the rigid endoscope was sterilized by high temperature and high pressure, and the flexible endoscope was sterilized with ethylene oxide. Each endoscope was sterilized once per day. A manual endoscopic traceable record was established.
3. Intervention C: from October 26–28, 2018, the outpatient cystoscope room was closed for 3 days, and complete terminal disinfection and sterilization of all endoscopes was carried out. Each flexible endoscope was used only once per day for one patient, and then sterilized with ethylene oxide.
4. Intervention D: the anesthetic gel product was recalled on November 26, 2018. After this point the outbreak was terminated.

## Results

### Epidemiological investigation

A total of 71 cases of *B. cepacia* nosocomial UTI were confirmed between March 19, 2018 and November 15, 2018. Among the 71 patients, there were 16 cases of *B. cepacia*-positive blood culture and 61 cases of *B. cepacia*-positive urine culture (6 cases of *B. cepacia*-positive blood and urine culture). The average patient age was 59 years, and 71.83% of patients were male. The time from exposure to the discovery of *B. cepacia* infection ranged from 0 to 65 days, with a median duration of 4 days. The majority of patients were from the urology (66.20%, 47/71) and gynecology (15.49%, 11/71) departments. The patients had a range of underlying conditions, including 47 cases of urinary tumors/stones/hydronephrosis, 11 cases of cervical cancer, and 11 cases with other illnesses accompanied by urinary disease. All of the patients required invasive diagnosis and urological treatment (examination/biopsies/placement of double-J stent, etc.) or bedside indwelling catheterization, and all equipment was lubricated with single-use anesthetic gel during intubation. Basic information on patients with *B. cepacia* UTI is provided in Table 1.

**Table 1:** Basic information on patients with *B. cepacia* UTI

Item	Case number (n = 71)
Age	59.21
Sex (male)	51
Underlying diseases n (%)	
Bladder tumor	25 (35.21)
Kidney stone or tumor	12 (16.90)
Ureter stone or tumor	10 (14.08)
Cervical cancer	11 (15.49)
Other diseases	11 (15.49)
Department distribution n (%)	
Urology surgery	47 (66.20)
Gynecology	11 (15.49)
Oncology	4 (5.63)
Other departments	9 (12.68)
Exposure operation	
Invasive operation of urology	66 (92.96)
Rigid endoscope	32 (45.07)
Flexible endoscope	20 (28.17)
Resectoscope	7 (10.61)
Unrecorded endoscope	7 (10.61)
Bedside catheterization	5 (7.04)
Antimicrobial therapy	
Quinolones	32 (45.07)
Carbapenems	18 (25.35)
Cephalosporins/others	11 (15.49)
Untreated	10 (14.08)
PFGE tested strains	20
PFGE homology strains	17

Totals may be unequal to 100% due to rounding

*IPC* infection prevention and control

**Fig. 1:**
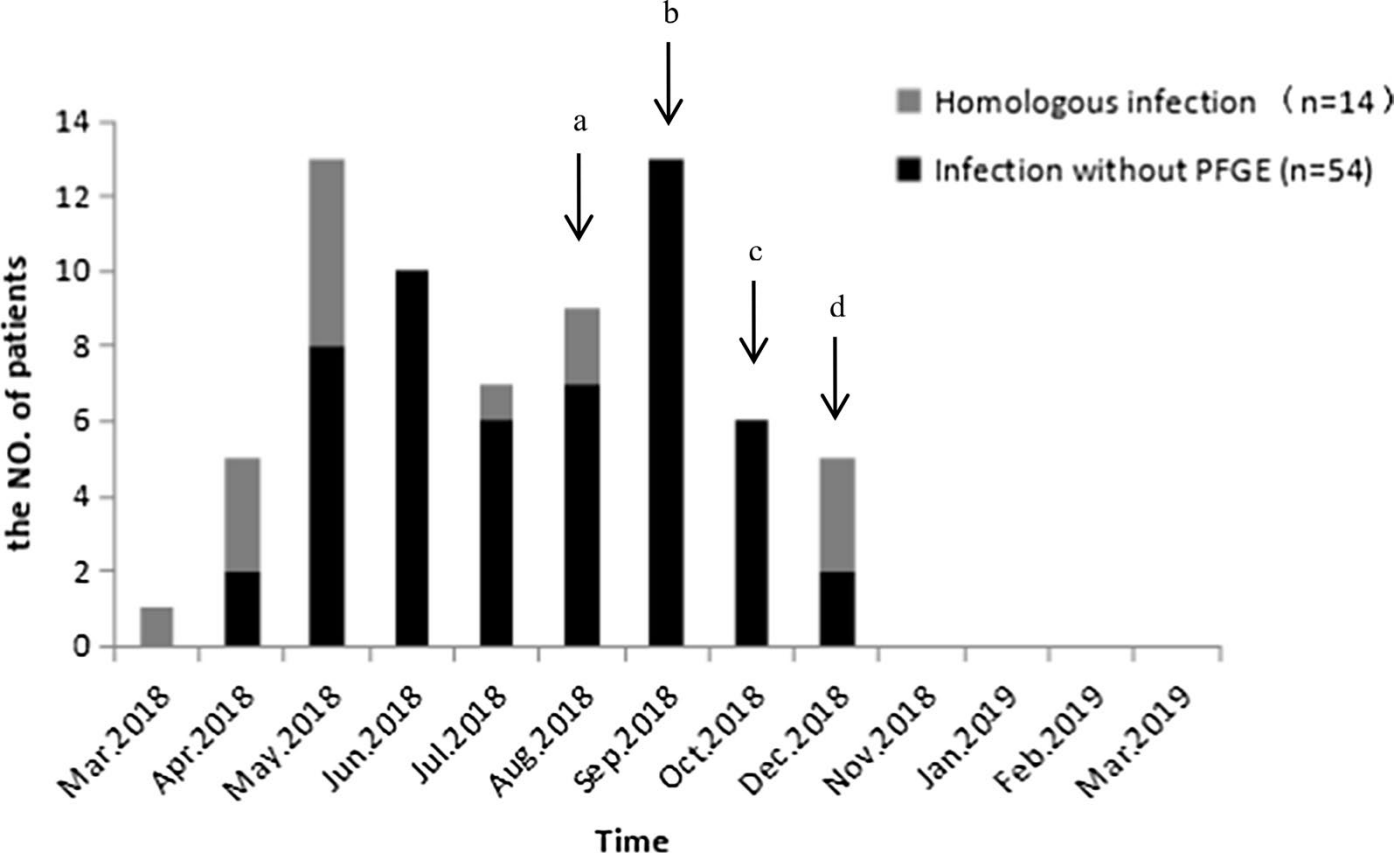
Epidemiological curve of the *B. cepacia* UTI outbreak. Even after implementing infection control measures, cases of infection continued to occur until the contaminated anesthetic gel was recalled and the outbreak terminated. **a** A report on August 21, 2018 showed that most *B. cepacia* infections originated prior to hospital admission, indicating that the cases did not constitute an outbreak. **b** On September 13, 2018, case findings were expanded to include the entire hospital and the epidemiological investigation was repeated, confirming the outbreak. *B. cepacia* was only detected from a cystoscope used by one patient. **c** Terminal disinfection was performed on October 26, 2018. **d** The cystoscope sampling method was replaced on November 11, 2018. *B. cepacia* was detected from one batch of anesthesia gel, then all anesthesia gels were recalled and the outbreak was terminated

A total of 71 patients with *B. cepacia* nosocomial UTI (positive blood and/or urine culture) were confirmed in 2018, and the epidemiological curves were developed. Two cases of non-nosocomial infection with positive blood cultures and five cases of non-nosocomial infection with positive urine cultures were excluded. The first case of infection occurred during diagnostic examination of a bladder tumor in the outpatient cystoscope room on March 13. The patient developed a fever and UTI after the procedure, and *B. cepacia* was detected in a urine sample on March 19. After the increased incidence of *B. cepacia* infection was reported in the Urology Surgery Department on August 21, three intervention measures were taken to gradually upgrade the cleaning and disinfection protocols for the urology endoscopes: first, cleaning and disinfection processes were strengthened; second, each endoscope was disinfected once per day, followed by daily sterilization with ethylene oxide; third, the flexible endoscope was used only once per day and then sterilized with ethylene oxide. However, the number of patients with *B. cepacia* infection continued to increase, including those who underwent procedures using the flexible and rigid endoscopes. On November 26, 2018, all batches of anesthesia gel used in the hospital were recalled and the outbreak abated. No new cases of infection occurred before the end of the monitoring period (31 March, 2019). All 71 patients were cured, and no deaths related to infection were recorded. The epidemic curve for the outbreak of infection is shown in Fig. 1.

### Microbiological identification

**Fig. 2:**
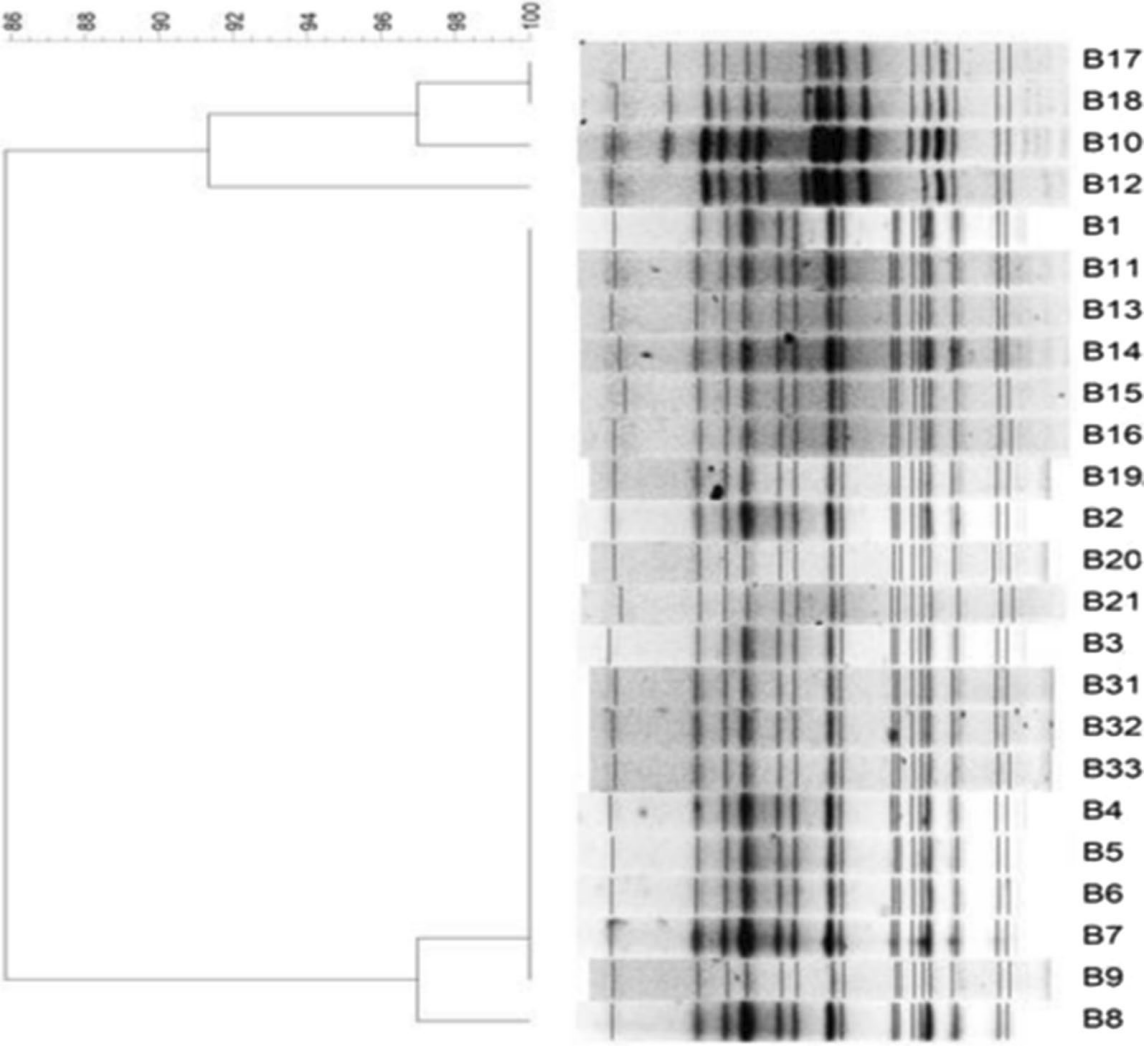
PFGE homology analysis of selected *B. cepacia* isolates. Isolates B1, B2, and B3 were recovered from anesthetic gel (20180701). Isolate B12 was recovered from a patient that acquired infection outside the hospital. The remaining 20 *B. cepacia* isolates were recovered from blood or urine samples from nosocomial UTI inpatients. Isolates B10 and B18 were both recovered from patients with bladder tumors accepted cystoscopy in the outpatient cystoscope room, and were detected by urine culture within 24 h of admission, corresponding to 15 and 65 days after cystoscopy, respectively. B17 was recovered from a patient with a ureter tumor who was diagnosed with *B. cepacia* infection 1 day after cystoscopy

A total of 155 microbiological samples were collected across four microbiological sampling points, including 42 samples from endoscopes, 38 from disposable items, 35 from environmental surfaces, 28 from instruments, eight from the hands and clothes of staff members, and four from water. Overall, *B. cepacia* was only detected in one used cystoscope sample and in one batch of anesthetic gel (20180701). The bacterial load in the anesthetic gel was 10^4^–10^5^ colony-forming units/ml. PFGE typing showed 98–100% homology between 17 *B. cepacia* isolates from patients and three isolates from the contaminated gel, although slight differences were observed among the three isolates from the gel (B1, B2, and B3). One isolate from a patient infected outside the hospital (B12) and three nosocomial infected *B. cepacia* isolates (B10,B17,B18) were non-homologous to the isolates from the anesthetic gel. The results of PFGE typing and analysis are shown in Fig. 2.

## Discussion

The current study reports an outbreak of *B. cepacia* UTI among 71 patients that was caused by contaminated single-use anesthetic gel. The investigation lasted for 4 months, microbiological sampling and identification were repeated, careful and detailed epidemiological investigation was carried out, and various intensive endoscope disinfection and sterilization interventions were implemented; however, infection cases continued to occur. Finally, the source of infection was determined, and the outbreak abated. Our experience in identifying and eliminating the source of the outbreak may be helpful as a reference for future outbreak situations.

When the increased incidence of *B. cepacia* infection was first reported in the Third Ward of the Urology Department, a preliminary epidemiological investigation was carried out. Nosocomial infection was defined only as *B. cepacia* identification later than 48 h post-admission. Therefore we wrongly ruled some nosocomial cases out. The case was defined as nosocomial *B. cepacia* UTI occurring after cystoscopy in the studied hospital regardless of the identification time. This finding suggests that during an investigation of an outbreak, investigators should carefully consult the patients and communicate with the clinical staff to understand the diagnosis and treatment process before and after infection.

*B. cepacia* was detected in a sample from a used, unsterilized cystoscope at the beginning of the investigation. We misjudged the source of *B. cepacia* infection as being contamination from the infected outpatient and inadequately sanitized cystoscope, but we ignored the possibility of contaminated disposable items. As such, subsequent microbial sampling only included the disinfected endoscope, resulting in a lost opportunity to identify the source of infection in the early stages. Therefore, when an outbreak involves equipment such as an endoscope, samples should be collected not only before use and after disinfection, but also immediately after use to determine whether pathogen contamination occurred during disinfection or in the process of diagnosis and treatment.

It is also important to determine the scope of the outbreak. We originally only included patients from the urology surgery ward, before expanding the investigation to include the entire hospital. We did not examine five difficult cases (7.04%, 5/71) requiring bedside indwelling catheterization. However, if the sources of exposure of these five patients had been studied early in the investigation, we would have been able to exclude the possibility of contamination arising from improper endoscope cleaning and disinfection of the outpatient cystoscope room, which would have suggested the anesthetic gel as the source of infection.

Outbreaks of nosocomial infection caused by *B. cepacia* often last for weeks or years [10, 11]. It is often very difficult to determine the source of infection, and the cause of the outbreak is often unknown. However, previous reports have traced *B. cepacia* outbreaks back to contaminated medical items, drugs, and hospital water sources used in diagnosis and treatment, while reports of *B. cepacia* outbreaks caused by person-to-person infection or endoscope cleaning and disinfection failures are rare [12]. Therefore, during nosocomial outbreaks of *B. cepacia* infection, investigations should focus on possible contamination of diagnosis and treatment equipment or items.

A review of the literature shows that there have been several nosocomial *B. cepacia* outbreaks in Europe and the United States [4, 6], but that the total number of cases in these outbreaks was small (< 20 cases). This may be because monitoring systems for nosocomial infection outbreaks have significantly improved in recent times, and infection control professionals and epidemiological experts are well equipped to investigate and control outbreaks. In addition, in the early stages of outbreak investigation, *B. cepacia* can be sent to regional or national joint investigation and control institutions where homology analysis or gene sequencing can be conducted quickly, and results can be compared with *B. cepacia* reference data in regional or national databases. The identification of identical *B. cepacia* strains in multiple hospitals is indicative of drug or equipment contamination, and national or regional recalls of the contaminated products can be undertaken once the source of contamination is confirmed [13].

However, because of the lack of monitoring and regional and/or national infection control facilities for nosocomial infections in developing countries, *B. cepacia* outbreaks are often only reported in one hospital. These outbreaks can last for a long time and affect many patients, meanwhile infected patients at other hospitals are very likely to be missed. All batches of anesthesia gel used in the hospital were recalled and we informed the gel manufacturer as soon as the contaminated gel confirmed. Before our incident, other hospitals also found that the infection outbreak or cluster was caused by the same reason [14]. The national medical products administration asked the manufacturer to stop production immediately and recall all the gels. But the manufacturer forgot to notify our hospital about the recall. Otherwise, we could have avoided this outbreak Contamination of water for production was the possible source of pollution due to B cepacia during the product manufacturing process. In 2016, *B. cepacia* was identified in blood samples from 14 patients across three intensive care units at a hospital in Saudi Arabia. The outbreak lasted for 6 months and was traced back to the use of contaminated ultrasound gel [15].

We acknowledge that this study had several limitations. First, although only one batch of anesthetic gel was contaminated with *B. cepacia*, the five batches were mixed and batch use was not recorded. As a result, it was not possible to track the total number of patients exposed to the contaminated anesthetic gel or the total number of infected patients. Second, there was no retrospective record of endoscopic diagnosis and treatment in the outpatient cystoscope room. Thus, the incidence of infection associated with different endoscopes could not be determined, making it difficult to investigate the outbreak. Third, because of the restrict of microbial resources, only partial *B. cepacia* specimens could be typed by PFGE.

## Conclusion

The internal monitoring and outbreak investigation capacity of hospitals in developing countries is insufficient, and many hospitals do not have access to technology such as microbiological homology identification. We suggest that each country should establish an effective national or regional laboratory platform for the prevention and control of infection outbreaks, allowing outbreak strains to be identified and compared against a national database. This system would help investigators to identify the source of infection and allow early intervention to terminate the outbreak.

## Data Availability

The datasets generated during the current study are not publicly available, to avoid disclosure of the individual privacy of the patients. However, they are available from the corresponding author on reasonable request.

## Abbreviations

UTI: urinary tract infection
PFGE: pulsed-field gel electrophoresis
